# One-Pot Preparation of HCPT@IRMOF-3 Nanoparticles for pH-Responsive Anticancer Drug Delivery

**DOI:** 10.3390/molecules28237703

**Published:** 2023-11-22

**Authors:** Hongda Cheng

**Affiliations:** Department of Pharmacy, Zibo Vocational Institute, Zibo 255300, China; chd_98766@163.com

**Keywords:** IRMOF-3, drug delivery, one pot, pH response, HCPT

## Abstract

Metal–organic frameworks (MOFs) are considered to be promising materials for drug delivery. In this work, a Zinc-based MOF nanocomposite IRMOF-3 was introduced as a drug carrier for 10-hydroxycamptothecine (HCPT). Without an extra drug-loading process, a nanoscale drug delivery material HCPT@IRMOF-3 was prepared via one-pot synthesis. The composition and structure of the material were investigated, and the drug release character was measured. Compared with preparing IRMOF-3 first and loading the drug, the one-pot-prepared HCPT@IRMOF-3 exhibited a higher drug-loading capacity. The material presented pH-responsive release. The HCPT release rate at pH 5.0 was significantly higher than that at pH 7.4. The cytotoxicity experiments showed that IRMOF-3 was non-toxic, and HCPT@IRMOF-3 exhibited notable cytotoxicity to Hela and SH-SY5Y cells. One-pot synthesis is a simple and rapid method for the preparation of an MOF drug delivery system, and IRMOF-3 can be potentially used in pH-responsive drug delivery systems.

## 1. Introduction

With changes in lifestyle and the deterioration of environmental pollution, cancer has become one of the most severe diseases faced by human beings. The number of cancer deaths worldwide has increased rapidly over the last decade, reaching approximately 9.6 million in 2018 [1]. As an essential treatment for cancer, drug therapy has been used widely in clinics. However, due to their poor solubility, the pharmacological function of some anticancer drugs is hampered, and the therapeutic effect is limited [2]. To overcome this deficiency, exploring nanoparticulate drug delivery systems to deliver hydrophobic drugs to cancer cells is an effective strategy. Nanocarriers can significantly promote the delivery of the drugs and improve their bioavailability, especially for drugs with poor water solubility. Some nanoparticles, such as polymers or mesoporous silica, have been developed as anticancer drug carriers [3,4,5,6]. However, the defects of their degradation resistance and low loading capacity limit their development. 

Metal organic frameworks (MOFs) are a kind of microporous solid adsorbent material which has been widely investigated and applied in the field of materials and chemistry in recent years. MOFs are constructed from metal nodes and organic linkers [7,8,9,10,11,12,13,14]. Metal ions such as di-, tri- or tetravalent metal ions form inorganic clusters in MOFs as coordination centers. Organic linkers, like phosphonates, carbohydrates and imidazolates, are interconnected between the metal nodes in one-, two-, or three-dimensional networks. The shape of MOFs is determined by the geometry of inorganic clusters and organic linkers and their connectivity. Compared with traditional porous adsorbent materials, MOFs exhibit great the advantages of excellent stability, a tunable pore size and a large surface area. Therefore, MOFs have attracted significant attention for many applications, such as catalysis, gas adsorption, magnetism, membrane separation, photonic, sensors and so on [15,16,17,18,19,20,21,22,23,24]. Owing to their large surface area, large pore volume and facile functionalization, MOFs are very suitable for use as biocompatible and biodegradable drug carriers, exhibiting great application potential in the field of drug delivery. MOFs can achieve efficient drug loading and controlled release due to the high porosity and facile surface functionalization. In recent decades, many MOFs have been explored as drug carriers. Jiang et al. synthesized Zr-based MOF ZJU-802, which was quite suitable for the oral drug delivery of anionic drugs. The drugs encapsulated in ZJU-802 could be easily released at pH 7.4, while they had a negligible release rate at pH 2.0 [25]. Gupta et al. synthesized nanoscale UiO-66 particles for loading docetaxel. The drug release results indicated that the release time of DTX@UiO-66 at higher pH was longer than that at a lower pH [26]. 

IRMOF-3 is one of the most popular MOFs, which has excellent thermal stability, high-level porosity and an ultra-large surface area [27]. IRMOF-3 has a structure of octahedral Zn_4_O(-COO)_6_ units. Four tetrahedral ZnO_4_ share a common vertex and combine with ligand BDC-NH_2_ to form a 3D framework structure. IRMOF-3 has important research and application potential because of the existence of an amino group. The electrostatic interaction and hydrogen bonds caused by the amino group can promote the dispersion of adsorbate in the material. Due to its excellent properties, IRMOF-3 exhibits promising application prospects in catalyst support, gas storage, sensing, drug delivery and energy storage [28,29]. 

For a drug delivery system, a separate drug-loading process seems inevitable, and the loading of the drug is complicated and time-consuming. In this study, we prepared a nanoscale metal–organic framework IRMOF-3 and used it as a carrier for anticancer drug 10-hydroxycamptothecine (HCPT). HCPT@IRMOF-3 was synthesized via the one-pot method, in which HCPT was directly added to the reaction mixture of IRMOF-3. The material was analyzed using XRD, SEM and TGA. The drug-loading capacity, drug release properties and cytotoxicity of the material were investigated.

## 2. Results and Discussion

### 2.1. Characterization of the Materials

**Figure 1:**
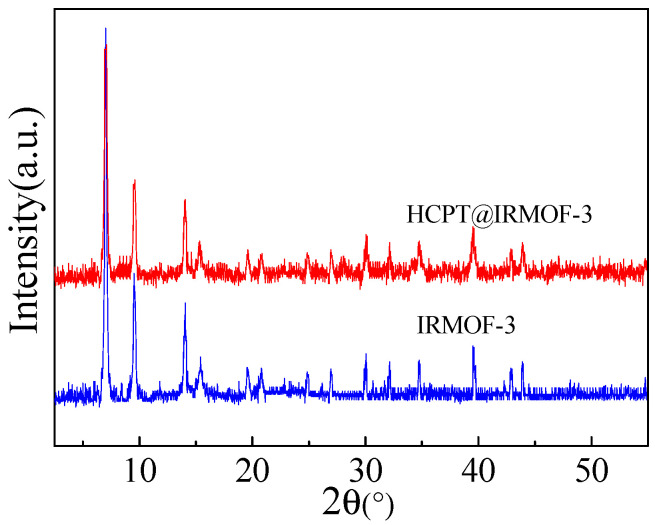
XRD patterns of IRMOF-3 and HCPT@IRMOF-3.

The powder X-ray diffraction (XRD) patterns of IRMOR-3 and HCTP@IRMOF-3 are shown in Figure 1. The diffraction peaks at 2θ = 6.9, 9.9, 13.8 in both cases are well defined, which confirms the highly crystalline nature of the material. The overall XRD pattern of IRMOF-3 matches well with the samples reported in the previous literature [30,31]. The XRD pattern of HCTP@IRMOF-3 is similar to that of IRMOF-3. The peak intensity of HCTP@IRMOF-3 is only slightly reduced at low angles, which may be due to the subtle effect of HCPT loading on the crystallinity of IRMOF-3. 

**Figure 2:**
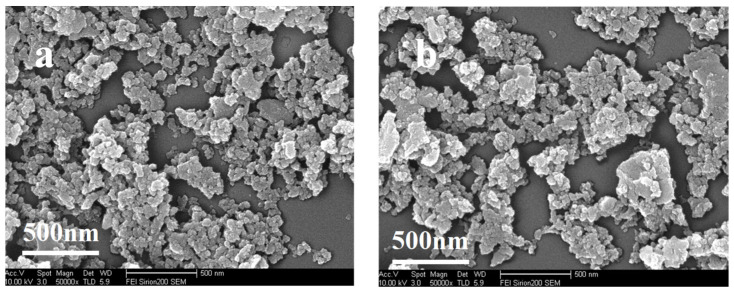
SEM images of IRMOF-3 (**a**) and HCPT@IRMOF-3 (**b**).

The morphologies of the synthesized IRMOF-3 and HCPT@IRMOF-3 were examined via SEM analysis. As can be seen from Figure 2, the morphologies of IRMOF-3 and HCPT@IRMOF-3 are almost the same, indicating that the embedding of the drug has a little effect on the morphology of IRMOF-3. The particle size of IRMOF-3 is estimated to be below 100 nm. The particle size and morphology are critical if MOFs are to be used as drug carriers. The carrier with particle size in the range of 10 to 100 nm is more effective for drug delivery purposes [32]. Due to the small size and increased surface area, nano-sized MOFs can effectively improve the pharmacodynamic properties of drugs.

**Figure 3:**
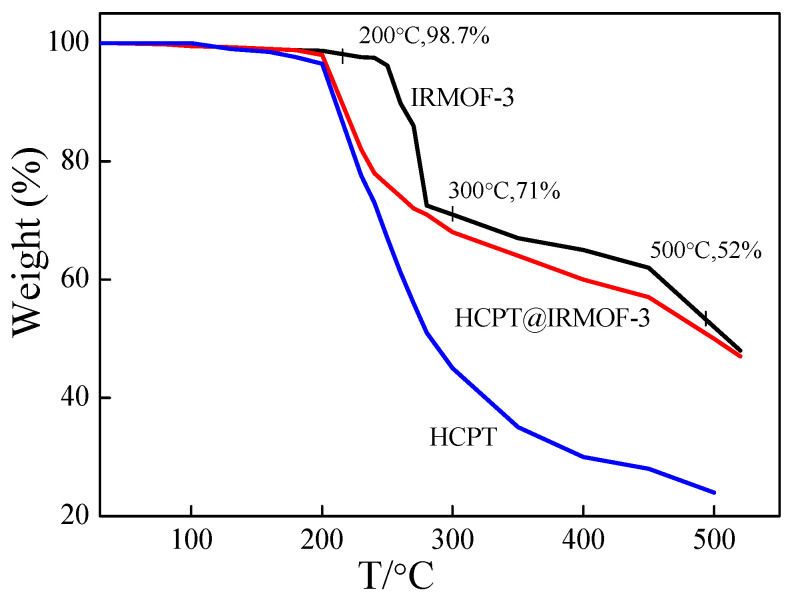
TGA analysis of IRMOF-3, HCPT and HCPT@IRMOF-3.

The thermal stability of nanomaterials plays an important role in their applications, especially in biomedical applications. The thermal degradation of IRMOF-3, HCPT@IRMOF-3 and HCPT were investigated via TGA at temperatures up to 500 °C, at a heating rate of 15 °C min^−1^ in an Ar atmosphere. As can be seen from Figure 3, IRMOF-3 remains stable with a mild change in the total mass of 3.8% until 260 °C. The weight loss is approximately due to the removal of chloroform or DMF, which is trapped in the pores of the material. A great weight loss is observed since 260 °C, which is related to the decomposition of the frameworks. At 500 °C, the weight loss reaches 48%, indicating a rupture in the main chains of the sample. Compared to IRMOF-3, the weight loss of HCPT@IRMOF-3 occurred earlier. Since the weight loss of HCPT begins at about 200 °C, the weight loss region of 200~300 °C of HCPT@IRMOF-3 may be related to the loss of HCPT. 

**Figure 4:**
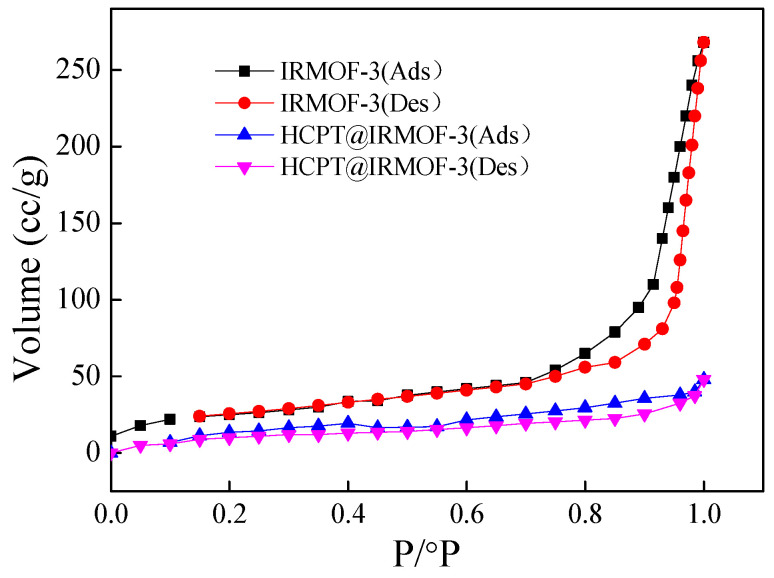
N_2_ adsorption–desorption isotherms of IRMOF-3 and HCPT@IRMOF-3.

The Nitrogen adsorption–desorption isotherms of IRMOF-3 were investigated. As shown in Figure 4, the N_2_ adsorption–desorption isotherms of IRMOF-3 demonstrate type IV isotherms with a hysteresis loop. The first half of the adsorption curve rises slowly and has an upward convex trend, indicating that the adsorption of IRMOF-3 transitions from single-molecular-layer adsorption to multimolecular-layer adsorption. The second half of the curve rises significantly, and there is no adsorption saturation near the saturate vapor pressure, indicating that the material has a mesoporous structure. The adsorption and desorption isotherms do not coincide, and a hysteresis loop is formed, indicating that the pore structure of the prepared IRMOF-3 is uniform. The N_2_ adsorption–desorption isotherm of HCPT@IRMOF-3 has a smaller hysteresis loop. The pore size of IRMOF-3 becomes smaller due to the loading of HCPT.

### 2.2. Drug Loading and Release of HCPT@IRMOF-3

**Figure 5:**
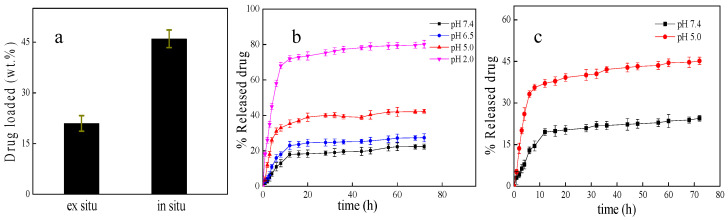
The drug-loading capacity of the materials, (**a**); pH-dependent drug release of HCPT@IRMOF-3 prepared by in situ method, (**b**); pH-dependent drug release of HCPT@IRMOF-3 prepared via ex situ method; (**c**). *n* = 3, mean ± SD.

10-hydroxycamptothecine (HCPT) is a natural antitumor alkaloid which has a good, curative effect on liver cancer, stomach cancer and bladder cancer. However, due to its poor solubility in water, low stability in physiological environment and high cytotoxicity, the clinical application of HCPT is limited. Loading HCPT with biocompatible nanoparticles can minimize these limitations. Therefore, IRMOF-3 was chosen as the drug carrier for HCPT. The drug-loading capacities of the materials were measured. As can be seen from Figure 5a, the HCPT loading capacity of HCPT@IRMOF-3 prepared via one-pot synthesis (in situ) reached 46 wt%, which is twice that of the two-step (ex situ) encapsulation route. The higher loading capacity may be attributed to the aggregation effect of Zn^2+^ on HCPT. Since the hydroxyl groups of HCPT can form coordination bonds with Zn^2+^, the HCPT molecules added to the reaction system were adsorbed around Zn^2+^, which makes HCPT easily encapsulated in the framework of IRMOF-3 when the material formed. 

The experiments show that the MOFs have different stabilities in different environments [33,34,35]. The stability of IRMOF-3 is related to the acidity of the environment. Therefore, the drug release of the substance should be related to the pH of the solution. The pH-responsive drug release of HCPT@IRMOF-3 under different pH conditions was investigated. As can be seen from Figure 5b,c, the drug release patterns of HCPT@IRMOF-3 prepared via in situ and ex situ methods are similar and can be divided into two parts. After an initial burst release during the first 12 h, HCPT@IRMOF-3 showed a sustained slow drug release pattern for 72 h. The burst release is attributed to the diffusion of drug molecules attached to the surface of IRMOF-3, and the subsequent sustained drug release may be ascribed to the release of drug molecules buried inside the pore of IRMOF-3. The drug release of HCPT@IRMOF-3 exhibited a significant pH response. The cumulative drug release rates at pH 7.4 were 22.4% (in situ) and 24.5% (ex situ) at 72 h. This release pattern is mainly related to the physical adsorption of HCPT by IRMOF-3, which is due to the large surface area and highly ordered porosity of IRMOF-3. When HCPT@IRMOF-3 was subjected in an acidified medium, pH = 5.0, a significant increase in HCPT release was detected, and the drug release capacity exceed 42.3% (in situ) and 45.6% (ex situ) at 72 h. Chemical bonding is a common method used to bind drugs to drug carriers [36]. Due to the presence of some special chemical groups such as -NH_2_, IRMOF-3 can combine with HCPT through a covalent bond or hydrogen bond. The nearly doubling of the drug release rate under an acidic pH may be due to the destruction of the chemical bond cooperation between IRMOF-3 and HCPT. The drug release of HCPT@IRMOF-3 at pH 2 reached 80%. This dramatic increase is probably caused by the decomposition of IRMOF-3. The coordination bond between zinc and amine was broken under acidic conditions. Therefore, the drug release of HCPT@IRMOF-3 exhibits a significant pH response, which makes it a potential pH-responsive drug delivery system for cancer treatment.

### 2.3. Cell Cytotoxicity Assay

**Figure 6:**
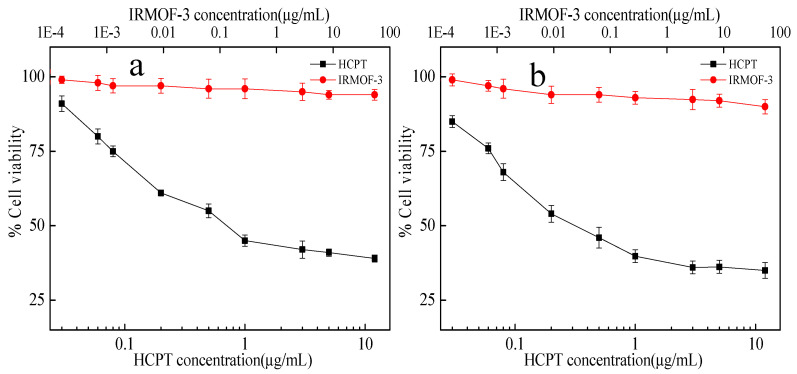
Cytotoxic effects of HCPT and IRMOF-3 in Hela cells exposed for 24 h (**a**) and 72 h (**b**).

**Figure 7:**
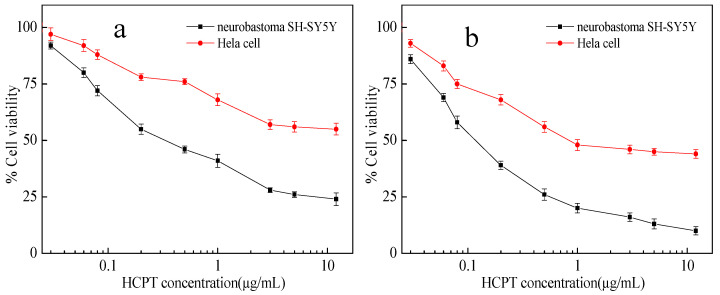
Cytotoxic effects of HCPT@ IRMOF-3 in Neuroblastoma SH-SY5Y and Hela cells exposed for 24 h (**a**) and 72 h (**b**).

The cytotoxic activity of the samples was validated via an MTT assay. Firstly, the biocompatibility of non-loaded material (IRMOF-3) and free HCPT over Hela cells was tested. As seen from Figure 6, the relative viability of Hela cells is above 90% even at the highest concentration of IRMOF-3, featuring IRMOF-3 as a strong candidate for drug loading with negligible cytotoxicity. The cell viability decreases with the increase in free HCPT. After incubation with 5 μg mL^−1^ HCPT for 24 and 72 h, the cell viabilities are 41% and 36%, respectively. Subsequently, the biocompatibility of HCPT@IRMOF-3 samples with a dose range of 0.001–10 μg mL^−1^ of equivalent HCPT over Hela and SH-SY5Y cell lines was tested (Figure 7). After incubation with HCPT@IRMOF-3 nanoparticles (containing 5 μg mL^−1^ HCPT) for 24 and 72 h, the cell viabilities of Hela are 56% and 45%, respectively, indicating that the cytotoxic activity level of HCPT@IRMOF-3 is lower than that of free HCPT. The half-maximal inhibitory concentration (IC_50_) values also approve of this result (Table 1). This may be due to the HCPT esterification over the nanoparticles. The enzymatic ester cleavage at the cytosol is limited, and it precludes the complete release of the drug.

## 3. Materials and Methods

### 3.1. Materials

All the reagents that used in the experiment were of analytical grade. 2-aminoterephthalic acid and zinc nitrate hexahydrate (Zn(NO_3_)_2_.H_2_O) were purchased from Sinopharm Shanghai Co., Ltd., Shanghai, China. *N*,*N*-dimethylformamide (DMF), dimethyl sulfoxide (DMSO), ethyl alcohol and chloroform were obtained from Aladdin. 3-(4,5-dimethylthiazol-2-yl)-2,5-diphenyltetrazolium bromide (MTT reagent) and phosphate-buffered solution (PBS) were purchased from Sigma-Aldrich. Hela and SH-SY5Y cells were purchased from Fenghui Biotechnology Co., Ltd., Changsha, China.

### 3.2. Preparation of HCPT@ IRMOF-3

HCPT-loaded IRMOF-3 (HCPT@IRMOF-3) was prepared via the one-pot method. Totals of 341 mg Zn (NO_3_)_2_·6H_2_O and 100 mg HCPT were first dissolved in 10 mL DMF, the solution was stirred for 1 h, and then 74 mg BDC-NH_2_ was added into the solution. The resulting mixture was transferred to a stainless steel reactor and heated for 20 h at 100 °C. After heating, the reactor was cooled to room temperature in air. The residual solvent was removed, and the product was washed with DMF (3 × 10 mL). Then, the product was dried by heating under vacuum oven at 120 °C for 10 h. UV-vis spectroscopy was used to measure the drug loading of the material at a wavelength of 254 nm. 

### 3.3. Preparation of IRMOF-3 and HCPT Loading

IRMOF-3 was synthesized via a solvothermal method, which is based on a previous technique with small modifications [27]. Totals of 74 mg BDC-NH_2_ and 341 mg Zn (NO_3_)_2_·6H_2_O were dispersed in 10 mL DMF and stirred to form a homogeneous solution at room temperature. Then, the solution was transferred to a stainless steel reactor and heated for 20 h at 100 °C. The residual solvent was removed, and the product was washed with DMF (3 × 10 mL) and CHCl_3_ (3 × 10 mL), respectively. Then, the product was dried via heating under a vacuum oven at 120 °C for 10 h.

As-synthesized IRMOF-3 was dispersed in 10 mL HCPT DMF solution (10 mg mL^−1^), which was mixed uniformly via ultrasound. The reaction occurred for 48 h, and then the solution was centrifuged at 5000 rpm for 10 min to obtain HCPT-capped IRMOF-3. The suspension was separated via filtration and washed with DMF (3 × 10 mL). 

### 3.4. Characterizations

The surface morphology and microstructure of the samples was investigated via scanning electron microscopy (SEM, FEISirion, Eindhoven, The Netherlands). An X-ray diffractometer (XRD, Bruker D8 Advance, Karlsruhe, Germany) was used to measure the phase compositions of the samples using Cu-Kα radiation with λ of 0.15404 nm. Thermo-gravimetric (TG, Linseis, TGA PT1000, Selb, Germany) analyses were performed in Ar at a heating rate of 15 °C min^−1^ from 30 to 600 °C. Nitrogen adsorption–desorption isotherms were acquired by using a gas adsorption analyzer (Micromeritics ASAP 2020) at −196 °C. The drug contents were measured with a UV-vis spectrometer (Shimadzu, Kyoto, Japan, UV-2700).

### 3.5. HCPT Release Study

A calibration plot of HCPT was first made before the drug release test. To investigate the drug release characteristics of the synthesized HCPT@IRMOF-3, the material was dispersed in 10 mL of phosphate-buffered saline (PBS) at 37 °C under four different pH conditions, pHs 2.0, 5.0, 6.5 and 7.4. The samples were incubated at 37 °C from 1 to 72 h, which were shaking in a water bath all the time. The samples were taken out periodically and centrifuged (12,000 r min^−1^). The supernatant PBS was collected and replaced with same amount of fresh PBS. The absorbance spectra of the PBS supernatants was measured, and the concentration of the drug in the sample was calculated by referring to the calibration plot. Then, the release percentages of HCPT were found using this formula: release percentage (%) = (weight of HCPT released by HCPT@IRMOF-3)/(weight of HCPT loaded in IRMOF-3).

### 3.6. Cytotoxicity Study

The in vitro cytotoxicities of IRMOF-3, HCPT@IRMOF-3 and free HCPT towards Hela and SH-SY5Y cells were assessed using a methyl thiazolyl tetrazolium (MTT) assay. The cells were seeded and stabilized for 24 h in 96-well plates (5000 cells per well). Then, the cells were incubated with various concentrations of IRMOF-3, HCPT@IRMOF-3 and free HCPT for 24 h or 72 h. The HCPT equivalents were 0.0002 to 20 μg mL^−1^. The cells were washed with PBS twice after incubation. MTT was added in each well and incubated for another 4 h. Thereafter, the formed formazan crystals were dissolved in DMSO, and the absorbance of each well at 595 nm was read using a microplate reader. The 50% inhibitory concentrations (IC50) were evaluated from the dose–response curve. A dose–response curve describes the relationship between response to drug treatment and drug concentration. The ordinate is the cell growth inhibition rate, and the abscissa is the drug concentration. The cell inhibitory rates of different concentrations of HCPT on cells were determined via MTT. The cell inhibitory rate = (A control − A experiment)/A control × 100%. A stands for the absorbance of the solution. Three independent experiments were performed for every sample, and all the data are expressed as mean ± standard error.

## 4. Conclusions

MOFs are promising materials for drug delivery due to their high porosity and facile surface functionalization. But there are a few studies on the use of MOFs as drug carriers, especially as carriers for anticancer drugs with poor water solubility. In this study, we prepared a nanoscale metal–organic framework IRMOF-3 and used it as a carrier for anticancer drug HCPT. HCPT@IRMOF-3 drug delivery nanomaterials were prepared using the one-pot method. Since HCPT was added directly to the reaction mixture, HCPT was encapsulated inside the carrier during the formation of the framework of IRMOF-3. According to the results of TG and nitrogen adsorption–desorption, the material has a good thermal stability and uniform mesoporous structure. The drug-loading capacity reaches 46 wt%, which is higher than those of most MOF materials reported until now. Under pH 5.0 and 7.4 conditions, the drug release percentages of the material in 72 h are 42.3% and 22.4%, respectively, indicating a significant pH response. These results confirm that one-pot method is suitable for the preparation and drug loading of IRMOF-3. IRMOF-3 is an excellent candidate for pH-responsive drug carriers.

## Figures and Tables

**Table 1:** Half-maximal inhibitory concentration of free HCPT and HCPT@IRMOF-3.

Cell Line	HCPT	HCPT@IRMOF-3
Hela	0.004 ± 0.001	0.061 ± 0.008
Neuroblastoma (SH-SY5Y)	8.94 × 10^−4^ ± 6.5 × 10^−5^	0.052 ± 4.3 × 10^−3^

## Data Availability

All details and data can be found in the text.
